# EGFR conjunct FSCN1 as a Novel Therapeutic Strategy in Triple-Negative Breast Cancer

**DOI:** 10.1038/s41598-017-15939-9

**Published:** 2017-11-15

**Authors:** Chao-Qun Wang, Yang Li, Bi-Fei Huang, Yong-Ming Zhao, Hui Yuan, Dongfang Guo, Chen-Ming Su, Gui-Nv Hu, Qian Wang, Tengyun Long, Yan Wang, Chih-Hsin Tang, Xiaoni Li

**Affiliations:** 1 Department of Pathology, Affiliated Dongyang Hospital of Wenzhou Medical University, Dongyang, 322100 Zhejiang, China; 2 Hefei National Laboratory for Physical Sciences at Microscale and School of Life Sciences, University of Science and Technology of China, 230027 Hefei, Anhui China; 3 Department of Surgical Oncology, Affiliated Dongyang Hospital of Wenzhou Medical University, Dongyang, 322100 Zhejiang, China; 4 Laboratory of Biomedicine, Affiliated Dongyang Hospital of Wenzhou Medical University, Dongyang, 322100 Zhejiang, China; 5 Department of Surgery, Anhui Medical University, 230027 Hefei, Anhui China; 6 Department of Medical Oncology, Affiliated Dongyang Hospital of Wenzhou Medical University, Dongyang, 322100 Zhejiang, China; 7 Graduate Institute of Basic Medical Science, China Medical University, 40402 Taichung, Taiwan; 8 Department of Pharmacology, School of Medicine, China Medical University, 40402 Taichung, Taiwan; 9 Department of Biotechnology, College of Health Science, Asia University, 40402 Taichung, Taiwan

## Abstract

Emerging evidence indicates that Fascin-1 (FSCN1) may possess a causal role in the development of several types of cancers and serves as a novel biomarker of aggressiveness in certain carcinomas. However, the regulatory mechanism of FSCN1 in triple-negative breast cancer (TNBC) cell invasion and migration is still largely unknown. In our study, we observed that the FSCN1 expression rates were significantly higher in invasive ductal carcinoma, compared with both usual ductal hyperplasia and ductal carcinoma *in situ*. FSCN1 expression was significantly higher in cases of TNBC compared with the non-TNBC subtype. Overexpression of FSCN1 promoted TNBC cell migration and invasion. Epidermal growth factor induced the expression of FSCN1 through activation of MAPK, which subsequently promoted cell migration and invasion. A significant decrease in FSCN1 expression following the co-treatment of FSCN1 siRNA and Gefitinib, compared with the separate treatment of FSCN1 siRNA or Gefitinib. Furthermore, we found that there was a significant association between FSCN1 expression and poor relapse-free survival and overall survival. Therefore, we suggest that co-targeting epidermal growth factor receptor and FSCN1 dual biomarker may be used as a novel therapeutic strategy for TNBC.

## Introduction

Triple-negative breast cancer (TNBC) is characterized by the absence of estrogen receptor (ER) and progesterone receptor (PR), as well as no over-expression of human epidermal growth factor receptor 2 (HER2), and is an aggressive subtype comprising 10–20% of breast cancer incidences^1–3^. Patients with TNBC have a shorter median survival time after relapse (18 months) and are more likely to develop chemo-resistant disease. In contrast to patients with ER/PR-positive or HER2-overexpressing tumors, TNBC remains a disease with poor prognosis and limited treatment options, and is not amenable to hormone therapy or HER2-targeting therapy, such as trastuzumab, and systemic treatment options are limited to cytotoxic chemotherapy^4–6^. Therefore, the development effective treatment strategies represent a pressing unmet clinical need.

Fascin-1 (FSCN1) is an actin-bundling protein, and its enhanced expression levels have been reported in several types of carcinomas, including that of the lung^7^, colon^8^, stomach^9^, ovary^10^, and breast^11^. In breast cancer, FSCN1 expression is associated with hormone receptor-negative, more aggressive clinical course, and also associated with TNBC in African American and Chinese women^11–13^. However, the mechanism underlying the effect of FSCN1 in the development of breast cancer, especially on TNBC, is yet to be elucidated.

In the present report, we used immunohistochemical (IHC) analysis to detect FSCN1 expression in a series of paraffin-embedded breast lesion tissue, including that from usual ductal hyperplasia (UDH), ductal carcinoma *in situ* (DCIS), and invasive ductal carcinoma (IDC), and analyzed the relationship between FSCN1 and TNBC or epidermal growth factor receptor (EGFR). The relationship between FSCN1 expression and clinical pathological features and prognosis were analyzed in patients with IDC. We further described the potential mechanism by which FSCN1 contributes to TNBC cell migration and invasion. In addition, we demonstrated that EGF induced the expression of FSCN1 through MAPK activation, subsequently promoting cell migration and invasion. Furthermore, we found there to be a significant decrease in FSCN1 expression following the co-treatment of FSCN1 siRNA and Gefitinib, compared with the separate treatment of FSCN1 siRNA or Gefitinib.

## Results

### FSCN1 is expressed in breast tissue specimens and is associated with clinical pathological parameters in patients with IDC

**Figure 1:**
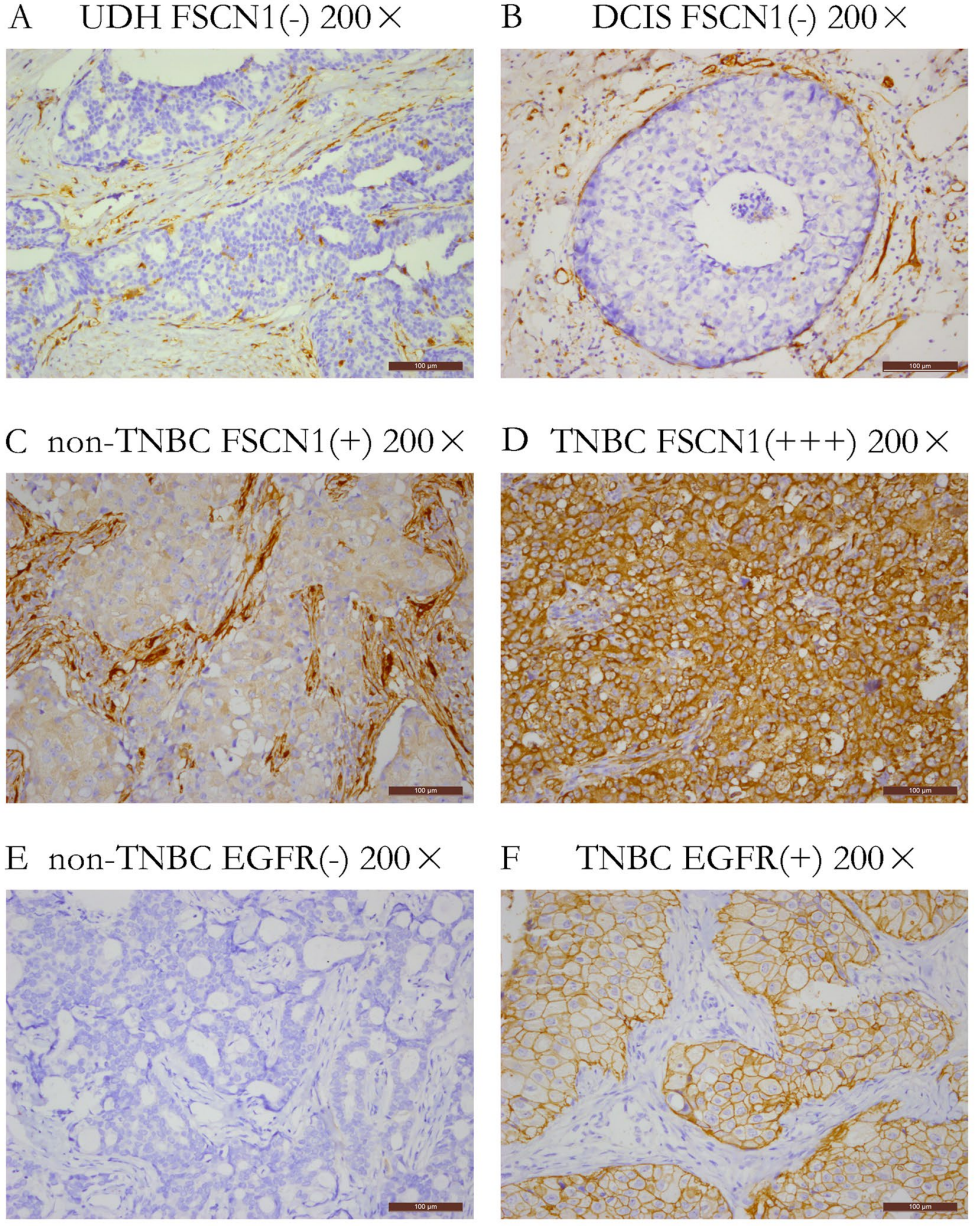
Immunochemical analysis of FSCN1 and EGFR expression in breast tissue specimens (magnification ×200). (**A**) UDH. FSCN1 expression is negative in proliferative ductal epithelial cells. (**B**) DCIS. FSCN1 expression is negative in intraductal cancer cells. (**C**) non-TNBC. Fascin-1 expression is positive in cancer cells. (**D**) TNBC. FSCN1 expression is strongly positive in cancer cells. (**E**) non-TNBC. EGFR expression is negative in cancer cells. (**F**) TNBC. EGFR expression is positive in cancer cells.

To define the potential role of FSCN1 in the progression of mammary carcinoma, we performed IHC analysis to assess the expression of FSCN1 on 125 UDH, 104 DCIS, and 467 IDC tissue samples (Fig. 1A–D). As shown in Table 1, the rates of positive FSCN1 expression in UDH, DCIS and IDC were 6.4% (8/125), 17.3% (18/104), and 33.0% (154/467), respectively (*P* < 0.0001). Additionally, we also observed that FSCN1 expression is significantly associated with a number of clinical parameters of IDC patients, including tumor size (*P* = 0.024), grade (*P* < 0.0001), stage (*P* = 0.045), ER- (*P* < 0.0001), PR- (*P* < 0.0001) and axillary lymph node metastasis (*P* = 0.024) (Table 2). Interestingly, we also observed that FSCN1 expression was significantly higher in cases of TNBC (88.6%, 62/70) compared with the non-TNBC subtype (19.2%, 71/370) (*P* < 0.0001) (Table 2), which is indicative that FSCN1 expression is associated with TNBC.

**Table 1:** The expression of FSCN1 in patients with UDH, DCIS, and IDC.

Group	No.	FSCN1 expression	
		Negative, n (%)	Positive, n (%)
UDH	125	117 (93.6%)	8 (6.4%)
DCIS	104	86 (82.7%)	18 (17.3%)
IDC	467	313 (67.0%)	154 (33.0%)*

Note: *P < 0.0001.

**Table 2:** Association of FSCN1 expression with clinical pathological parameters in IDC patients.

Parameter	No.	FSCN1 Expression, n (%)	P-value
**Age (years)**			
≤35	21	9 (42.9%)	0.610
35–55	288	93 (32.3%)	
>55	158	52 (32.9)	
**Tumor size (cm)**			
≤2	235	66 (28.1%)	0.024
2–5	209	76 (36.4%)	
>5	23	12 (52.2%)	
**Lymphnode metastases**			
−	235	66 (28.1%)	0.024
+	232	88 (37.9%)	
**Tumor grade**			
I	27	3 (11.1%)	0.000
II	329	82 (24.9%)	
III	111	69 (62.2%)	
**Tumor stage**			
I	140	35 (25.0%)	0.045
II	208	73 (35.1%)	
III	119	46 (38.7%)	
IV	0	0 (0.00)	
**Estrogen receptor**			
−	177	113 (63.8%)	0.000
+	290	41 (14.1%)	
**Progesterone receptor**			
−	223	121 (54.3%)	0.000
+	244	33 (13.5%)	
**c-erbB-2 expression**			
0–1+	203	76 (37.4%)	0.045
2+	154	39 (25.3%)	
3+	110	39 (35.5%)	
**Molecular classification**			
non-TNBC subtype	370	71 (19.2%)	0.000
TNBC subtype	70	62 (88.6%)	

### FSCN1 expression is associated with TNBC

**Figure 2:**
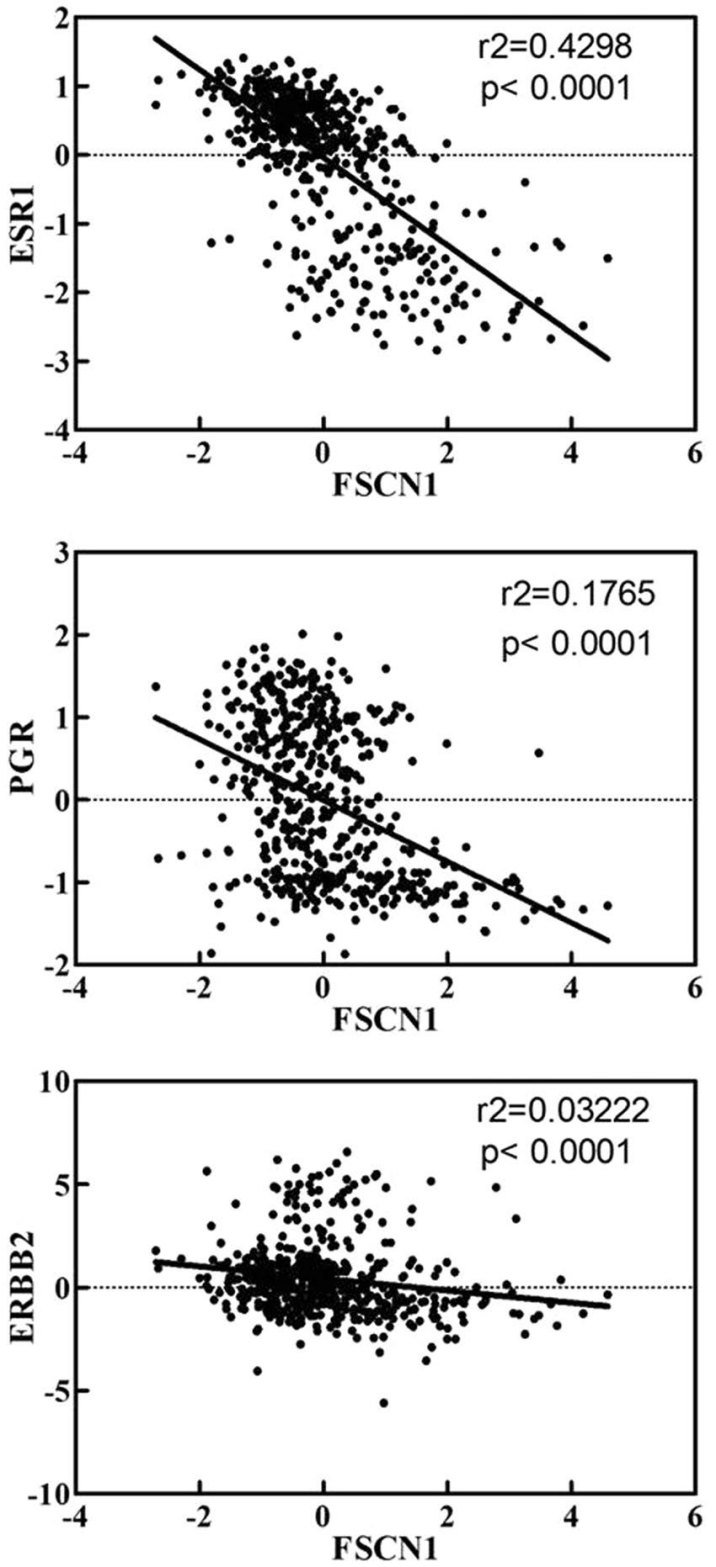
Correlation analysis of the TCGA Breast Invasive carcinoma database (TCGA, Provisional 2012) using cBioPortal showed the correlation between ESR1 (ER)/PGR (PR)/ERBB2 (Her2) and FSCN1 mRNA levels.

To determine the association between FSCN1 expression and ER, PR, HER2, respectively, we performed an integrative analysis of mRNA expression of clinical data published by The Cancer Genome Atlas (TCGA) database. As shown in Fig. 2, we found significantly negative correlation between FSCN1 and ER, PR, and HER2 (*P* < 0.0001), further suggesting that FSCN1 expression is associated with TNBC.

### FSCN1 promotes migration and invasion of TNBC *in vitro*

**Figure 3:**
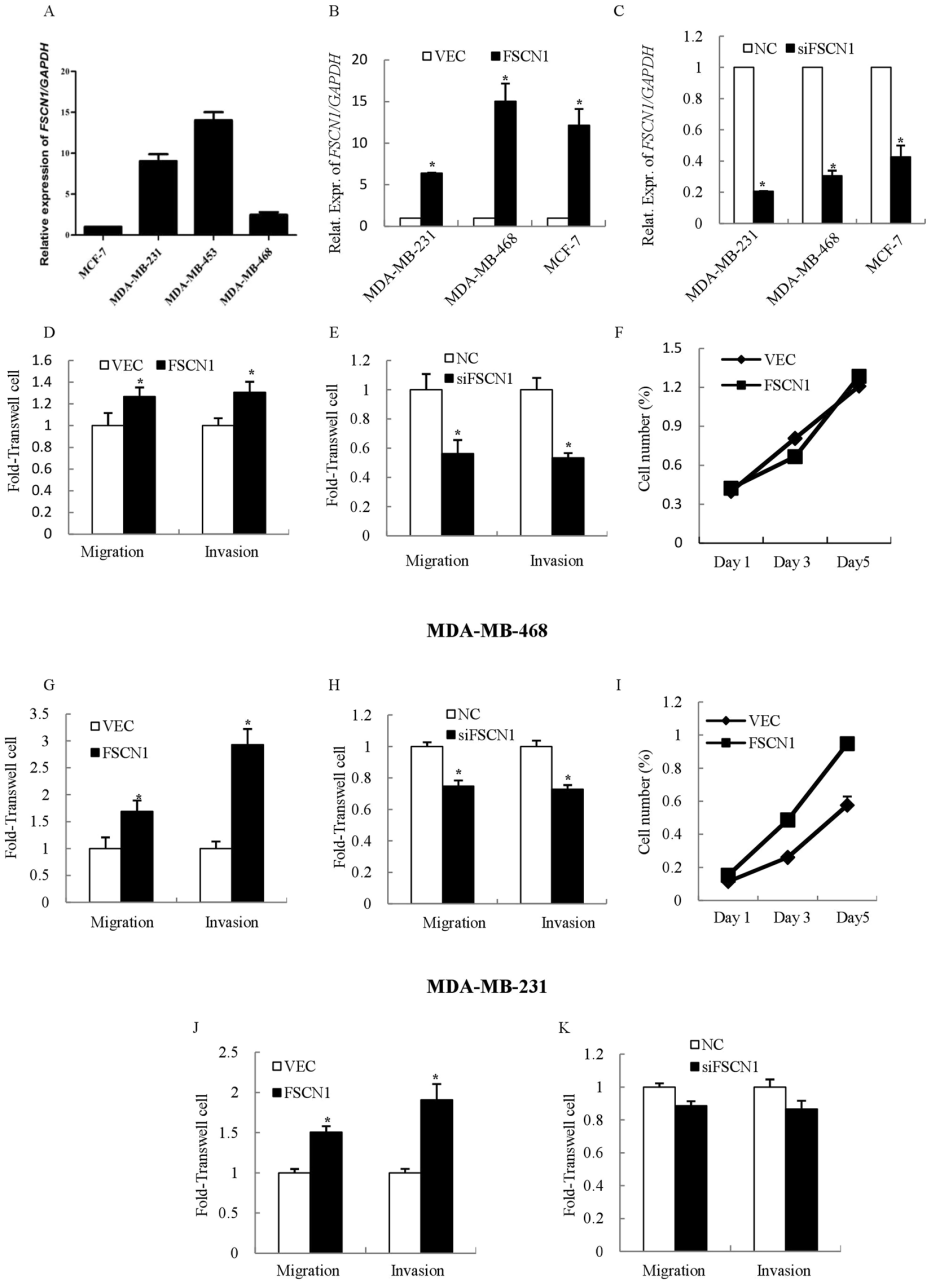
FSCN1 promotes TNBC migration and invasion *in vitro*. (**A**) Expression levels of FSCN1 in four mammary carcinoma cell lines were analyzed using real-time PCR. FSCN1 expression was normalized to GAPDH. The data are presented as mean ± S.E.M. from 3 independent experiments, each performed in triplicate. (**B**) Expression levels of FSCN1 in MDA-MB-231, MDA-MB-468 and MCF-7 cell lines transfected with FSCN1 plasmid or vector as a negative control were analyzed using real-time PCR. (**C**) Expression levels of FSCN1 in MDA-MB-231, MDA-MB-468 cell and MCF-7 lines transfected with FSCN1 small interfering RNA (siRNA) or the control were analyzed using real-time PCR. D-K. FSCN1 modulates substantial migration, invasion, and growth in MDA-MB-468 (**D**,**E**,**F**), MDA-MB-231 (G, H, I) and MCF-7 (J, K) cell lines. Cells were grown and transiently transfected with FSCN1 plasmid or vector as a negative control for 2 days and subjected to Transwell (**D**,**G**,**J**) and MTT (**F**,**I**) assays. Cells were grown and transiently transfected with FSCN1 small interfering RNA (siRNA) or the control for 2 days and subjected to Transwell assays (**E**,**H**,**K**). Values are technical triplicates (were performed independently three times) and represent mean ± S.E.M. *P < 0.01.

In order to further understand the possible role of FSCN1 in TNBC, we next assessed the potential impact of FSCN1 on mammary carcinoma cell migration and invasion in TNBC cells. We performed a preliminary screen for expression levels of FSCN1 in four mammary carcinoma cell lines (contains one of the non-TNBC cell line MCF-7, and three TNBC cell lines MDA-MB-468, MDA-MB-231 and MDA-MB-453) by Real-Time Polymerase Chain Reaction (PCR) (Fig. 3A). Then, we determined the potential impact of FSCN1 on the phenotype of the TNBC cells MDA-MB-468 and MDA-MB-231 migration and invasion, through manipulation of the expression levels of FSCN1 by transfection of FSCN1 expression plasmids (Fig. 3B). As shown in Fig. 3, forced expression of FSCN1 resulted in significantly increased MDA-MB-468 (Fig. 3D) and MDA-MB-231 (Fig. 3G) cells migration and invasion compared with control vector. Conversely, depletion of FSCN1 by transfection of FSCN1 siRNA resulted in a significant reduction in cell migration and invasion compared with cells transfected with control oligonucleotides in the MDA-MB-468 and MDA-MB-231 cell lines (Fig. 3C,E,H). Furthermore, forced expression of FSCN1 resulted in significantly increased non-TNBC MCF-7 cell migration and invasion compared with control vector (Fig. 3B,J). In comparison, there was no significant reduction of migration and invasion in the MCF-7 cell line which express very low level of FSCN1, compared with cells transfected with control oligonucleotides (Fig. 3C,K). Taken together, these data indicate that overexpression of FSCN1 dramatically promotes invasiveness of TNBC cells *in vitro*.

It is notable that FSCN1 expression was able to influence proliferation of MDA-MB-231 cells; however, this was not the case for the MDA-MB-468 cell line (Fig. 3F,I). In consolidation with these results, a previous report^7^ also demonstrated that FSCN1 promoted the migration and invasive ability, but not the proliferation of non-small cell lung cancer. This suggested that FSCN1 has various functional roles in a highly context-dependent manner in different cell lines.

### EGF regulates the FSCN1 expression and activates MAPkinase signaling in TNBC

We further investigated the signaling pathway modulated by FSCN1 in the progression of TNBC. EGF has previously been demonstrated to regulate MAPK activation^14^. We and others have also previously reported that both EGF and MAPK enhanced breast cancer cell migration and invasion^15,16^. Therefore, we further sought to determine whether FSCN1 contributed to EGF-mediated mammary carcinoma cell migration and invasion.

**Figure 4:**
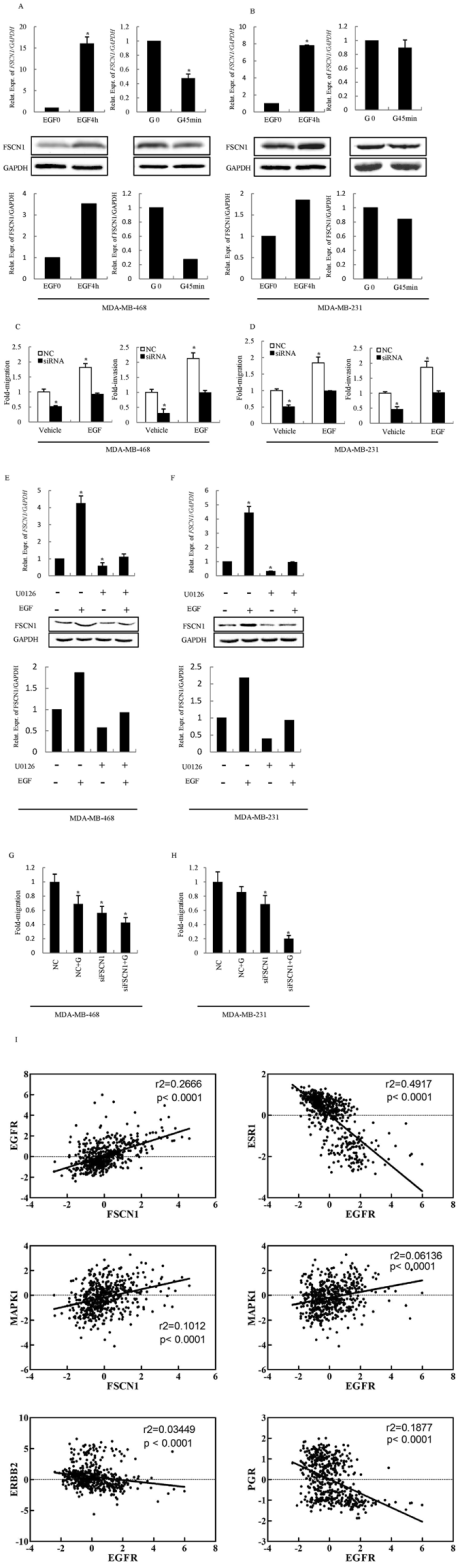
EGF regulates FSCN1 expression and activates MAP kinase signaling in TNBC. (**A**,**B**) Expression levels of FSCN1 mRNA and protein in the MDA-MB-468 (**A**) and MDA-MB-231 (**B**) cell lines treated with EGF/Gefitinib. Upper, Real-time PCR validated the expression levels of FSCN1 mRNA in the MDA-MB-468 and MDA-MB-231 cell lines. FSCN1 expression is normalized to GAPDH. The data are presented as the mean ± S.E.M. from three independent experiments, each performed in triplicate. *P < 0.01. Under, Western blot was used to analyze the expression levels of FSCN1. Total cellular protein was isolated and subjected to western blot analysis for FSCN1 expression. GAPDH was used as loading control. Bottom, The quantitative result of western blot is shown. (**C**,**D**) Transwell assays. Cells were grown and transfected with FSCN1 siRNA or a control siRNA for 2 days. Serum deprived cells were sebsequently treated with 20 ng/mL EGF or with a Vehicle for 4 h. Cells were subsequently subjected to assays. *P < 0.01. (**E**,**F**). Expression levels of FSCN1 mRNA and protein in the MDA-MB-468 (**E**) and MDA-MB-231 (**F**) cell lines treated with EGF/U0126. Upper, Real-time PCR validated the expression levels of FSCN1 mRNA in the MDA-MB-468 and MDA-MB-231 cell lines. FSCN1 expression is normalized to GAPDH. The data are presented as the mean ± S.E.M. from three independent experiments, each performed in triplicate. *P < 0.01. Under, Western blot was used to analyze the expression levels of FSCN1. Total cellular protein was isolated and subjected to western blot analysis for FSCN1 expression. GAPDH was used as loading control. Bottom, The quantitative result of western blot is shown. (**G**,**H**) Transwell assays. Cells were grown and transfected with FSCN1 siRNA or control siRNA for 2 days and treated with 100 ng/mL Gefitinib or vehicle for 45 min. Cells were subsequently subjected to assays. *P < 0.01. I. Correlation analysis of the TCGA Breast Invasive carcinoma database (TCGA, Provisional 2012) using cBioPortal show the association between MAPK1/EGFR and FSCN1 mRNA levels, in addition to ESR1 (ER)/PGR (PR)/ERBB2 (Her2)/MAPK1 and EGFR mRNA levels. *P* < 0.0001.

We first demonstrated that mRNA and protein expression levels of FSCN1 in EGF-stimulated TNBC cells were significantly increased. Conversely, the treatment of TNBC cells with EGFR inhibitor Gefitinib^17^, resulted in decreased mRNA and protein expression of FSCN1 in the MDA-MB-468 cell line, but not in the MDA-MB-231 cell line (Fig. 4A,B, Supplementary Figure 1A,B), suggesting that there is likely to be Gefitinib resistance in TNBC cells. Transfection of FSCN1 siRNA consistently decreased EGF regulated cell migration and invasion (Fig. 4C,D), indicating that EGF stimulated TNBC cell function modulated by the combinatory functionality of FSCN1.

To further determine whether MAPK was required for FSCN1 mediated TNBC cell migration and invasion, we assessed FSCN1 expression levels by co-treatment cells with EGF and MAPK specific inhibitor U0126. As shown in Fig. 4E,F, inhibition of MAPK activity was demonstrated to diminish the mRNA and protein expression levels of FSCN1 while EGF promoted that of FSCN1(Supplementary Figure 1C,D). Moreover, MAPK specific inhibitor U0126 abrogated the enhancement of FSCN1 expression stimulated by the treatment with EGF. It is therefore apparent that EGF/MAPK signaling was utilized to drive invasion and migration consequent to forced expression of FSCN1.

Importantly, we detected a significant decrease in migration after co-treatment with FSCN1 siRNA and Gefitinib, compared to the single treatment with FSCN1 siRNA and Gefitinib (Fig. 4G,H), suggesting that combined inhibition of EGFR and FSCN1 may be used as a novel therapeutic strategy for TNBC.

Furthermore, we analyzed the association between EGFR expression and ER, PR, and HER2 using TCGA database. Interestingly, we found a significantly negative correlation between EGFR and ER, PR, and HER2 (*P* < 0.0001). Consistently, we found there was positive correlation between FSCN1 and EGFR, MAPK1 (*P* < 0.0001, Fig. 4I). Thus, we hypothesized that FSCN1 utilizes the EGFR-MAPK signaling pathway to play functionality.

Additionally, we detected the expression of EGFR in IDC using IHC and analyzed its relationship with TNBC. EGFR expression levels were significantly higher in TNBC (78.6%, 55/70) compared with non-TNBC (44.3%, 164/370) (*P* < 0.0001, Table 3, Fig. 1E,F), and FSCN1 expression was significantly higher in EGFR positive IDC cases (51.6%, 126/243) compared with EGFR negative cases (12.5%, 28/224) (*P* < 0.0001, Table 4).

**Table 3:** The expression of EGFR in TNBC and non-TNBC patients.

Group	No.	EGFR expression	
		Negative, n (%)	Positive, n (%)
TNBC	70	15 (21.4%)	55 (78.6%)*
non-TNBC	370	206 (55.7%)	164 (44.3%)

Note: *P < 0.0001.

**Table 4:** The relationship between EGFR and FSCN1 in patients with IDC.

Group	No.	FSCN1 expression	
		Negative, n (%)	Positive, n (%)
EGFR (+)	243	117 (48.1%)	126 (51.9%)*
EGFR (−)	224	196 (87.5%)	28 (12.5%)

Note: *P < 0.0001.

### FSCN1 expression is associated with survival of patients with IDC

To assess the potential impact of FSCN1 on patient survival, we analyzed FSCN1 expression in relation to relapse-free survival (RFS) and overall survival (OS) in patients with IDC.

**Figure 5:**
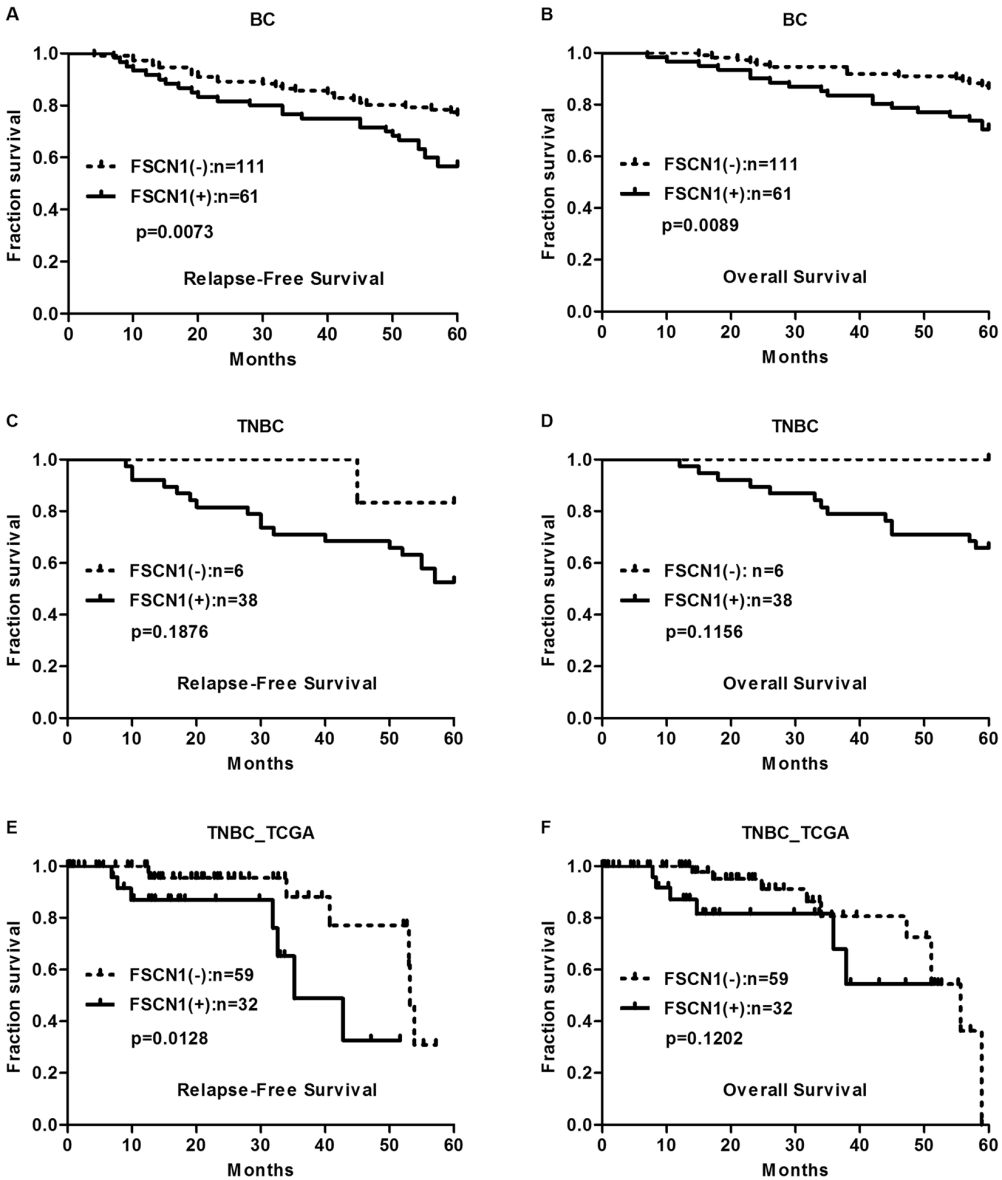
FSCN1 expression was associated with the survival of patients with IDC. (**A**,**B**) The association of FSCN1 expression with relapse-free survival (RFS) (**A**), overall survival (OS) (**B**) of the IDC patients were analyzed. (**C**,**D**) The association of FSCN1 expression with relapse-free survival (RFS) (**C**), overall survival (OS) (**D**) of the TNBC patients were analyzed. (**E**,**F**) Kaplan-Meier curves for relapse-free survival (RFS) (**E**), overall survival (OS) (**F**) of low and high expression of FSCN1 mRNA in TNBC subtype of breast cancer. Data was extracted from TCGA. P values were calculated with Log-rank (Mantel-Cox) test.

As shown in Fig. 5A,B, patients whose primary tumors expressing FSCN1 (n = 61) had a mean RFS of 47.5 months (at a 55.7% 5-year RFS rate), whereas patients whose tumors did not express FSCN1 (n = 111) had a mean RFS of 53.2 months (at a 76.6% 5-year RFS rate, *P* = 0.0073, Fig. 5A). Patients whose primary tumors expressed FSCN1 (n = 61) had a mean OS of 52.6 months (at a 70.5% 5-year OS rate), whereas patients who did not express FSCN1 (n = 111) had a mean OS of 57.1 months (at a 86.5% 5-year OS rate, *P* = 0.0089, Fig. 5B). Taken together, these results suggested that FSCN1 could be used as a potential clinical biomarker for breast cancer.

We further specifically analyzed the survival in TNBC cases. As shown in Fig. 5C,D, FSCN1 positive patients were associated with worse RFS and OS in our TNBC cases, which was consistent with our finding in the IDC patients. However, no unequivocal association was observed in our 44 TNBC cases (*P* = 0.1876 and *P* = 0.1156, respectively). Considering that there were only 6 FSCN1 negative cases in our collection, we further analyzed 91 TNBC patients from 1105 samples of TCGA breast cancer database. TNBC patients were divided into two groups based on median FSCN1 mRNA expression of all patients, and subsequently RFS and OS were further analyzed. As shown in Fig. 5E,F, we analyzed and found that high expression of FSCN1 was significantly associated with worse 5-year RFS, while there was no unequivocal association was observed between FSCN1 and OS. Taken together, these results suggested that high FSCN1 could be potentially used as a predictor of disease recurrence in patients with TNBC.

## Discussion

Reports concerning FSCN1 as a novel therapeutic biomarker for aggressive and metastatic carcinomas are limited^18–21^. In this study, we observed that the expression levels of FSCN1 were significantly higher in patients with IDC, compared with those in patients with UDH and DCIS. In addition, FSCN1 expression was associated with a number of poor prognostic characteristics in patients with IDC, suggesting that FSCN1 may be linked to the progression of mammary carcinoma. In addition, we also observed that FSCN1 expression was significantly higher in TNBC compared with the non-TNBC subtype, and we verified the potential functional role of FSCN1 expression in the progression of TNBC. Therefore, our findings assist in understanding the functional role of FSCN1 in TNBC progression, and may provide new understanding of the mechanism of neoplastic progression. FSCN1 is expected to possess other functional roles in addition to the findings detailed herein. For example, knockdown or knockout of FSCN1 can lead to a reduction of nodal signal transduction and endoderm formation in zebrafish embryos, in addition, the depletion of FSCN1 disrupts the association between receptors and actin filaments and sequesters the internalized receptors into clathrin-coated vesicles^22^.

TNBC is an aggressive subtype associated with poor survival, and unlike patients with the non-TNBC subtype, TNBC is not amenable to hormonal or HER2-targeting therapy^4–6^. Therefore, the identification of novel treatment strategies for TNBC is important. In this study, we found that FSCN1 expression was significantly higher in TNBC and dramatically promotes invasiveness of TNBC cells *in vitro*. Then, we sought to identify how FSCN1 promotes TNBC invasion. EGFR, is initially expressed on the plasma membrane in an inactive form, and becomes activated through certain kinases and/or after binding to its specific ligands^23–26^. It is known that EGFR activation occurs via kinases and/or transactivation through binding to specific ligands, including EGF^27^. EGFR is abnormally expressed and activated in many cancer cells, and initiates signal transduction cascades that promote cell division, migration and angiogenesis^28^. We and others have shown that EGFR is highly expressed in TNBC and is a potential therapeutic target, and is associated with poor prognosis^29–31^.

At present, EGFR inhibitor Gefitinib and anti-EGFR monoclonal antibody cetuximab, have shown little efficacy in the majority of clinical studies regarding TNBC^32–35^. Drug resistance to EGFR targeted therapy is caused by a variety of reasons, including MET amplification and overexpression^36^ and nuclear EGFR^37^. Therefore, for the identification of therapeutic targets that act synergistically with EGFR targeted therapies has potential to improve the survival rate and quality of life of patients with TNBC. Our results showed that the EGFR/MAPK/FSCN1 signaling pathways promotes TNBC invasion and a greater inhibitory effect on TNBC cells was observed after the co-treatment of FSCN1 siRNA with Gefitinib compared with the separate treatment of FSCN1 siRNA or Gefitinib. We also observed decreased FSCN1 expression and migration ability in MDA-MB-468 cells after treatment with Gefitinib, but these effects were not obvious in MDA-MB-231 cells. And we found that FSCN1 expression in MDA-MB-231 cells was significantly higher than that in MDA-MB-468 cells. Thus, we hypothesized that TNBC cells exhibit resistance to EGFR targeted therapy, part of the mechanism may be that EGFR/MAPK cannot completely regulate FSCN1, and there may be other upstream regulatory mechanisms affecting FSCN1. Taken together, our discovery indicates that co-targeting EGFR and FSCN1 dual biomarker could significantly decrease the abilities of migration and invasion in TNBC.

It should be noted that FSCN1 could be expected to possess a wide range of functions in the progression of breast cancer. The functions of FSCN1 are yet to be fully elucidated. Further studies are therefore required to fully understand the functional roles and interactions of FSCN1 with regard to therapeutics for breast cancer, particularly for TNBC.

## Methods and Materials

### Patients and specimens

The female patient population consisted of 467 women with breast cancer, 104 patients with DCIS and 125 patients with UDH. All specimens were obtained from the Tissue Bank of the Affiliated Dongyang Hospital of Wenzhou Medical University (Zhejiang, China) between 2007 and 2014. All patients had been undergoing primary surgical treatment and did not receive any preoperative treatment. Pathohistological diagnosis was made based on the breast tumor classification criteria of the World Health Organization^38^. Histology grade was determined in accordance with the Scarff-Bloom-Richardson system^39^. We obtained follow-up information from the 172 patients with breast cancer, and the median follow-up time was 62 months (range, 7–70 months). The Institutional Review Board of Affiliated Dongyang Hospital of Wenzhou Medical University approved this study, and informed consent forms were signed by patients or their guardians. All methods were performed in accordance with the relevant guidelines and regulations by University of Science and Technology of China and Affiliated Dongyang Hospital of Wenzhou Medical University.

### IHC Analysis

IHC staining of paraffin-embedded tissue sections was carried out using the Envision System (Dako, Glostrup, Denmark) as described previously^13^. The primary antibodies used were as follows: anti-FSCN1 mouse monoclonal antibody (clone 55k-2, diluted at 1:100, Santa Cruz Biotechnology, Santa Cruz, USA); anti-EGFR rabbit polyclonal antibody (clone 1005, diluted at 1:100, Santa Cruz Biotechnology); HercepTest (Dako); ready-to-use anti-ER rabbit monoclonal antibody (clone SP1, Dako); ready-to-use anti-PR mouse monoclonal antibody (clone PR636, Dako).

### FISH Analysis

Breast cancer cases with HER2 2+ equivocal results determined by IHC analysis required further detection using FISH. In this study, there were 27 cases of breast cancer with ER-, PR-, and HER2 2+ equivocal status did not undergo FISH, and leads to uncertain classification as TNBC or non-TNBC subtype. FISH analysis was performed on paraffin embedded tissue sections using the PathVysion HER2 DNA Probe Kit (Abbott-Vysis, Des Plaines, Illinois) as described previously^13^.

### Assessment of Staining

The entire tissue section was scanned and scored separately by two pathologists for assessment of staining. Staining intensity and extent were recorded in cancer cells to assess of FSCN1 expression. Staining intensity was scored as 0 (negative), 1 (weak), 2 (medium), or 3 (strong); Staining extent was scored as 0 (0%), 1 (1–25%), 2 (26–50%), 3 (51–75%), or 4 (76–100%); Sum of staining intensity and extent scores ≥3 and percentage of invasive tumor cells with cytoplasmic staining >5% were considered to be positive for FSCN1. Invasive tumor cells with ≥10% membrane staining were considered to be positive for EGFR. A case was considered to be ER or PR positive if the percentage of positive invasive cancer cells (nuclear staining) was >5%^11^. HER2 status was assessed according to the 2013 American Society of Clinical Oncology/College of American Pathologists (ASCO/CAP) guidelines for HER2 testing in breast cancer^40^.

### Cell culture

All human breast cancer cell lines used in this study were obtained from the American Type Culture Collection (Rockville, MD) and cultured in conditions as recommended. All cells were maintained in a humidified incubator at 37 °C and 5% CO_2_.

### Reagents

Media, sera and antibiotics for cell culture were purchased from Life Technologies, Inc. (Grand Island, NY, USA). Protein electrophoresis reagents were from Bio-Rad Laboratories (Richmond, VA, USA). Gefitinib was purchased from Tocris Bioscience (Ellisville, MO, USA). All other chemicals were from Sigma-Aldrich (St Louis, MO, USA).

### Transfections (siRNA)

Cells were transfected with FSCN1 siRNA or their respective negative controls (GenePharma, Shanghai) using Lipofectamine2000 (Invitrogen) following the manufacturer’s protocol. Cells were harvested in TRIzol (Invitrogen) for RNA extraction and in RIPA lysis buffer for protein extraction.

### Transfections (plasmid)

Cells were transfected with FSCN1 plasmid or vector using effectene (Qiagen) following the manufacturer’s protocol. Cells were harvested in TRIzol for RNA extraction and in RIPA lysis buffer for protein extraction.

### Transwell migration and invasion assay

Assays were performed in BioCoat Matrigel invasion chambers (Corning Costar, Acton, MA) as described previously^41^. Values for cell migration or invasion were expressed as the average number of cells per microscopic field. All experiments were performed at least three times.

### Cell proliferation assay

A cell count kit-8 (CCK-8) (Kumamoto Techno Research Park, Japan) was used to examine cell proliferation. CCK-8 includes WST-8 [2-(2-methoxyl-4-nitrophenyl)-3-(4-nitrophenyl)-5-(2,4-disulfonicacid benzene)-2H-tetrazalium sodium] that can be reduced to a highly water-soluble formazan dye, which is yellow. In brief, cells were incubated with 100 mL of culture medium in 96-multiwell plates. Media were removed and 100 mL F12 containing CCK-8 (10%) was added to each well. After 2 h incubation at 37 °C, the absorbance of each well was measured at 450 nm using a standard enzyme-linked immunosorbent assay (ELISA)-format spectrophotometer. The experiments were performed in triplicate and repeated thrice.

### RNA and Western Blot analysis

These procedures were carried out as described previously^15^. Membranes were blocked with 5% milk powder in PBS and then incubated with: anti-FSCN1 (clone 55k-2, diluted at 1:1000, Santa Cruz Biotechnology), anti-GAPDH (clone 2A8, diluted at 1:5000, Abmart, Shanghai, China).

### Plasmid constructs

The FSCN1 sequence was amplified with primers as follows:

Forward-ATGATTCTCGAGCCTCGCTCTGGGAGTACTAGGG,

Reverse-TATATATGCGGCCGCTGGGGCTGCAGACTGAGTTATT, and was cloned into the HindIII and XhoI sites at the multiple cloning regions of the pcDNA3.1 plasmid.

### Database analysis

The gene expression profiling of 825 invasive breast carcinomas (with recorded progression stages) was generated by TCGA database. Among those samples, 299 had no information regarding serous grades. Our analysis was based on the Agilent microarray (TCGA, Nature 2012). The Kaplan-Meier survival curve analyses of 1105 samples (TCGA, Provisional) were carried out using log-rank tests in GraphPad Prism (GraphPad Software). TNBC patients were divided into two groups based on median FSCN1 mRNA expression of all patients, and subsequently RFS and OS were further analyzed.

### Statistical analysis

All statistical analyses were performed using SPSS 19.0 software (SPSS Inc, Chicago, IL, USA). Numerical data are expressed as mean ± standard error of the mean from a representative experiment performed in triplicate. Differences/correlations between groups were compared using Pearson’s chi-square test for qualitative variables and Student’s t-test for continuous variables. Patient RFS and OS rates were analyzed using the Kaplan-Meier method and compared by log-rank analysis. *P* < 0.05 was considered statistically significant.

## Electronic supplementary material

Supplementary Information

## References

[1] De Laurentiis M (2010). Treatment of triple negative breast cancer (TNBC): current options and future perspectives. Cancer treatment reviews.

[2] Peddi PF, Ellis MJ, Ma C (2012). Molecular basis of triple negative breast cancer and implications for therapy. International journal of breast cancer.

[3] Boyle P (2012). Triple-negative breast cancer: epidemiological considerations and recommendations. Annals of oncology: official journal of the European Society for Medical Oncology/ESMO.

[4] Li H (2012). An essential role of metalloprotease-disintegrin ADAM12 in triple-negative breast cancer. Breast cancer research and treatment.

[5] Dent R (2007). Triple-negative breast cancer: clinical features and patterns of recurrence. Clinical cancer research: an official journal of the American Association for Cancer Research.

[6] Haffty BG (2006). Locoregional relapse and distant metastasis in conservatively managed triple negative early-stage breast cancer. Journal of clinical oncology: official journal of the American Society of Clinical Oncology.

[7] Zhao J (2010). Upregulated fascin1 in non-small cell lung cancer promotes the migration and invasiveness, but not proliferation. Cancer letters.

[8] Jawhari AU (2003). Fascin, an actin-bundling protein, modulates colonic epithelial cell invasiveness and differentiation *in vitro*. The American journal of pathology.

[9] Hashimoto Y, Shimada Y, Kawamura J, Yamasaki S, Imamura M (2004). The prognostic relevance of fascin expression in human gastric carcinoma. Oncology.

[10] Hu W (2000). *Increased expression of fascin, motil*ity associated protein, in cell cultures derived from ovarian cancer and in borderline and carcinomatous ovarian tumors. Clinical & experimental metastasis.

[11] Yoder BJ (2005). The expression of fascin, an actin-bundling motility protein, correlates with hormone receptor-negative breast cancer and a more aggressive clinical course. Clinical cancer research: an official journal of the American Association for Cancer Research.

[12] Esnakula AK (2014). Strong association of fascin expression with triple negative breast cancer and basal-like phenotype in African-American women. Journal of clinical pathology.

[13] Wang CQ (2016). Fascin-1 as a novel diagnostic marker of triple-negative breast cancer. Cancer medicine.

[14] van Cruijsen H, Giaccone G, Hoekman K (2005). Epidermal growth factor receptor and angiogenesis: Opportunities for combined anticancer strategies. International journal of cancer.

[15] Li X (2013). c-MYC-regulated miR-23a/24-2/27a cluster promotes mammary carcinoma cell invasion and hepatic metastasis by targeting Sprouty2. The Journal of biological chemistry.

[16] Jiang X (2004). Formation of tissue factor-factor VIIa-factor Xa complex promotes cellular signaling and migration of human breast cancer cells. Journal of thrombosis and haemostasis: JTH.

[17] Woodburn JR (1999). The epidermal growth factor receptor and its inhibition in cancer therapy. Pharmacol Therapeut.

[18] Tan, V. Y., Lewis, S. J., Adams, J. C. & Martin, R. M. Association of fascin-1 with mortality, disease progression and metastasis in carcinomas: a systematic review and meta-analysis. *Bmc Med* **11**, 10.1186/1741-7015-11-52 (2013). 10.1186/1741-7015-11-52 PMC3635876 23442983

[19] Kulasingam, V. & Diamandis, E. P. Fascin-1 is a novel biomarker of aggressiveness in some carcinomas. *Bmc Me*d **1**1, 10.1186/1741-7015-11-53 (2013). 10.1186/1741-7015-11-53 PMC3626678 23443037

[20] Hashimoto Y, Kim DJ, Adams JC (2011). The roles of fascins in health and disease. J Pathol.

[21] Hashimoto Y, Skacel M, Adams JC (2005). Roles of fascin in human carcinoma motility and signaling: Prospects for a novel biomarker?. Int J Biochem Cell B.

[22] Liu Z (2016). Fscn1 is required for the trafficking of TGF-beta family type I receptors during endoderm formation. Nature communications.

[23] Olayioye MA, Neve RM, Lane HA, Hynes NE (2000). The ErbB signaling network: receptor heterodimerization in development and cancer. The EMBO journal.

[24] Schlessinger J (2000). Cell signaling by receptor tyrosine kinases. Cell.

[25] Yarden Y, Sliwkowski MX (2001). Untangling the ErbB signalling network. Nature reviews. Molecular cell biology.

[26] Tao RH, Maruyama IN (2008). All EGF(ErbB) receptors have preformed homo- and heterodimeric structures in living cells. Journal of cell science.

[27] Wang X (2016). Sphingosine 1-Phosphate Activation of EGFR As a Novel Target for Meningitic Escherichia coli Penetration of the Blood-Brain Barrier. PLoS pathogens.

[28] Jorissen RN (2003). Epidermal growth factor receptor: mechanisms of activation and signalling. Experimental cell research.

[29] Park HS (2014). High EGFR gene copy number predicts poor outcome in triple-negative breast cancer. Modern pathology: an official journal of the United States and Canadian Academy of Pathology, Inc.

[30] Sutton LM (2010). Intratumoral expression level of epidermal growth factor receptor and cytokeratin 5/6 is significantly associated with nodal and distant metastases in patients with basal-like triple-negative breast carcinoma. American journal of clinical pathology.

[31] Corkery B, Crown J, Clynes M, O’Donovan N (2009). Epidermal growth factor receptor as a potential therapeutic target in triple-negative breast cancer. Annals of oncology: official journal of the European Society for Medical Oncology/ESMO.

[32] Carey LA (2012). TBCRC 001: randomized phase II study of cetuximab in combination with carboplatin in stage IV triple-negative breast cancer. Journal of clinical oncology: official journal of the American Society of Clinical Oncology.

[33] Baselga J (2013). Randomized phase II study of the anti-epidermal growth factor receptor monoclonal antibody cetuximab with cisplatin versus cisplatin alone in patients with metastatic triple-negative breast cancer. Journal of clinical oncology: official journal of the American Society of Clinical Oncology.

[34] Baselga J (2005). Phase II and tumor pharmacodynamic study of gefitinib in patients with advanced breast cancer. Journal of clinical oncology: official journal of the American Society of Clinical Oncology.

[35] von Minckwitz G (2005). A multicentre phase II study on gefitinib in taxane- and anthracycline-pretreated metastatic breast cancer. Breast cancer research and treatment.

[36] Sohn J (2014). cMET Activation and EGFR-Directed Therapy Resistance in Triple-NegativeBreast Cancer. Journal of Cancer.

[37] Brand TM (2013). Nuclear EGFR as a molecular target in cancer. Radiotherapy and oncology: journal of the European Society for Therapeutic Radiology and Oncology.

[38] Lakhani, S. R., E. I., Schnitt, S. J., Tan, P. H. & van de Vijver, M. J. WHO Classification of Tumours of the Breast, Fourth Edition. *Lyon: IARC Press* (2012).

[39] Elston CW, Ellis IO (1991). Pathological prognostic factors in breast cancer. I. The value of histological grade in breast cancer: experience from a large study with long-term follow-up. Histopathology.

[40] Wolff AC (2013). Recommendations for human epidermal growth factor receptor 2 testing in breast cancer: American Society of Clinical Oncology/College of American Pathologists clinical practice guideline update. Journal of clinical oncology: official journal of the American Society of Clinical Oncology.

[41] Chen J (2011). CCL18 from tumor-associated macrophages promotes breast cancer metastasis via PITPNM3. Cancer cell.

