# Supplementary material for: EGFR conjunct FSCN1 as a Novel Therapeutic Strategy in Triple-Negative Breast Cancer
Source: Sci Rep. 2017 Nov 15;7:15654. doi: 10.1038/s41598-017-15939-9 (PMC5688137; doi:10.1038/s41598-017-15939-9)
Supplement: Supplementary file 1 — Supplementary Information [file 41598_2017_15939_MOESM1_ESM.pdf]

EGFR conjunct FSCN1 as a Novel Therapeutic Strategy in Triple-Negative Breast Cancer

Chao-Qun Wang[1 §], Yang Li[2 §], Bi-Fei Huang[1], Yong-Ming Zhao[3], Hui Yuan[2], Dongfang Guo[2], Chen-Ming Su[4], Gui-Nv Hu[3], Qian Wang[1], Tengyun Long[5], Yan Wang[6], Chih-Hsin Tang[7-9]& Xiaoni Li[2*]

[1]Department of Pathology, Affiliated Dongyang Hospital of Wenzhou Medical University, Dongyang, Zhejiang, China 322100

[2]Hefei National Laboratory for Physical Sciences at Microscale and School of Life Sciences, University of Science and Technology of China, Hefei, Anhui, China 230027

[3]Department of Surgical Oncology, Affiliated Dongyang Hospital of Wenzhou Medical University, Dongyang, Zhejiang, China 322100

[4]Laboratory of Biomedicine, Affiliated Dongyang Hospital of Wenzhou Medical University, Dongyang, Zhejiang, China 322100

[5]Department of Surgery, Anhui Medical University, Hefei, Anhui, China 230027

[6]Department of Medical Oncology, Affiliated Dongyang Hospital of Wenzhou Medical University, Dongyang, Zhejiang, China 322100

[7]Graduate Institute of Basic Medical Science, China Medical University, Taichung, Taiwan 40402

[8]Department of Pharmacology, School of Medicine, China Medical University, Taichung, Taiwan 40402

[9]Department of Biotechnology, College of Health Science, Asia University, Taichung, Taiwan 40402

[§]Contributed equally

[*]**Corresponding author:** Xiaoni Li, Ph.D.

Hefei National Laboratory for Physical Sciences at Microscale and School of Life Sciences

University of Science and Technology of China

443 Huang Shan Road, Hefei 230027, China

Phone: +86-0579-86856765

Fax: +86-0579-86856878

E-mail: XIL183@pitt.edu

Supplementary Figure

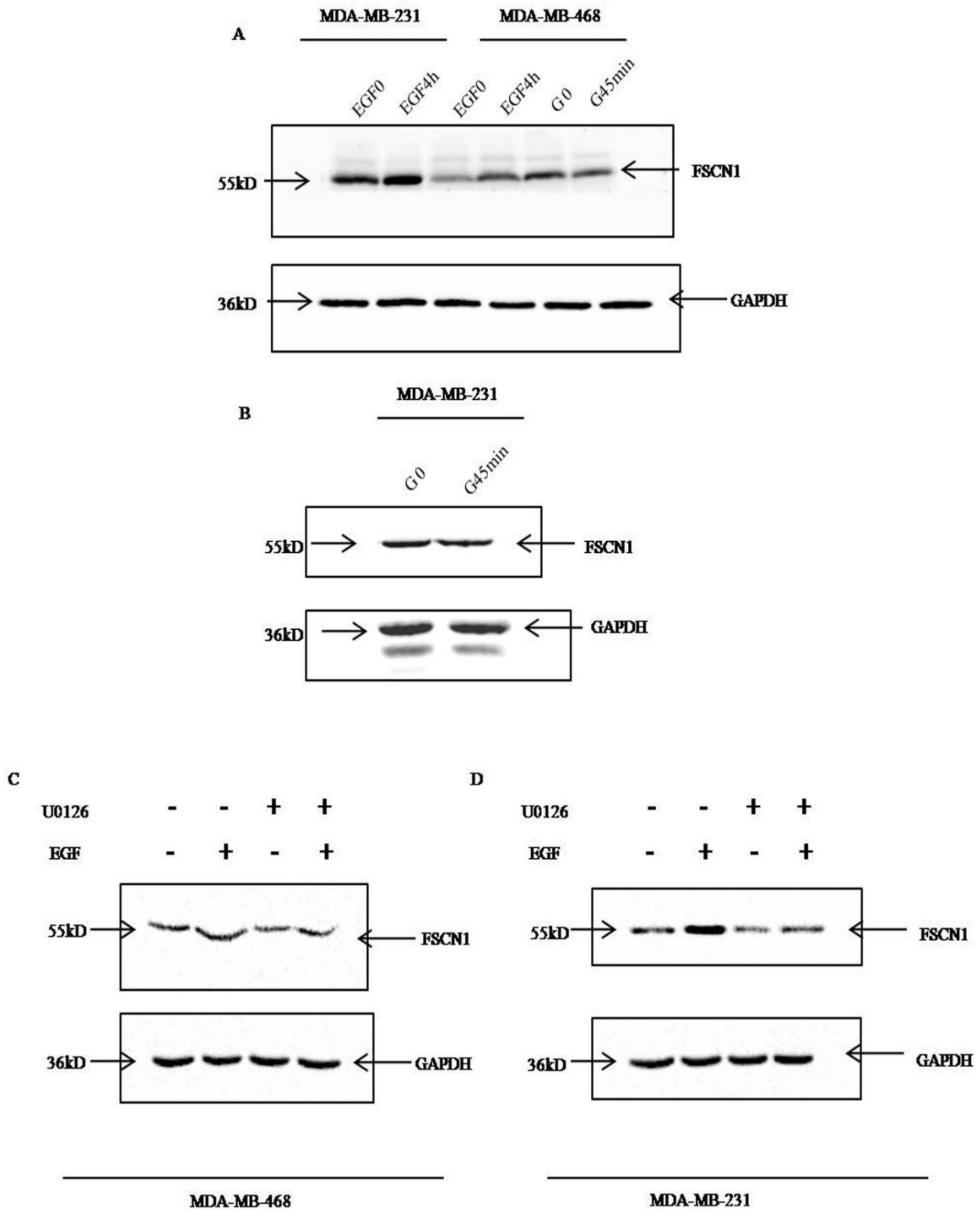

Figure S1. Uncropped blots for Western blot.

(A, B). Full-length immunoblots of Figure 4A and 4B.

C. Full-length immunoblots of Figure 4E.

D. Full-length immunoblots of Figure 4F.
